# A comparative analysis of clinicopathological features and survival between pre and postmenopausal breast cancer from an Indian cohort

**DOI:** 10.1038/s41598-023-30912-5

**Published:** 2023-03-09

**Authors:** Vidya P. Nimbalkar, Savitha Rajarajan, Snijesh V P, Annie Alexander, Rohini Kaluve, Sumithra Selvam, Rakesh Ramesh, Srinath B S, Jyothi S. Prabhu

**Affiliations:** 1 Division of Molecular Medicine, St. John’s Research Institute, St. John’s Medical College, Bangalore, Karnataka India; 2 Centre for Doctoral Studies, Manipal Academy of Higher Education (MAHE), Manipal, Karnataka India; 3 Division of Epidemiology, Biostatistics and Population Health, St. John’s Research Institute, St John’s Medical College, Bangalore, Karnataka India; 4 Department of Surgical Oncology, St. John’s Medical College and Hospital, Bangalore, Karnataka India; 5 Department of Surgery, Sri Shankara Cancer Hospital and Research Centre, Bangalore, Karnataka India

**Keywords:** Oncology, Risk factors

## Abstract

Breast cancer (BC) among premenopausal women is an aggressive disease associated with poor outcome despite intensive treatment. Higher burden is observed in southeast Asian countries attributed to younger population structure. We compared the reproductive and clinicopathological characteristics, distribution of subtypes and survival between pre and postmenopausal women from a retrospective cohort of BC patients with median follow up over 6 years to examine the differences. In our cohort of 446 BC patients, 162/446 (36.3%) were premenopausal. Parity and age at last childbirth were significantly different between pre and postmenopausal women. Premenopausal BC had a higher proportion of HER2 amplified and triple negative breast cancer (TNBC) tumors (*p* = 0.012). Stratified analysis by molecular subtypes showed TNBC had significantly better disease free (DFS) and overall survival (OS) among premenopausal group (mean survival, pre vs. post, DFS = 79.2 vs. 54.0 months, OS = 72.5 vs. 49.5 months, *p* = 0.002 for both). Analysis on external datasets (SCAN-B, METABRIC) confirmed this finding for overall survival. Our data confirmed the previously observed association of clinical and pathological features between pre and postmenopausal BC. Exploration of better survival among premenopausal TNBC tumors is warranted in larger cohorts with long term follow up.

## Introduction

Breast cancer (BC) is the most common cancer worldwide. Though incidence is higher in developed nations, mortality due to BC is significantly higher in less developed countries^1^. As per the national cancer registry program (2020), the incidence of BC is rising and now is the most diagnosed cancer among women within India^2^. Women with BC in India are a decade younger in comparison to western counterparts, thus affecting younger women in premenopausal age group^3,4^. Previous studies have reported young women have higher mortality even if diagnosed early and receive an intense treatment compared to the women in the elderly age group^5–7^. BC in the young women tends to be more aggressive and are often diagnosed in the advanced stage of the disease and have unfavorable tumor characteristics like larger tumor size, lymph node positivity^5,7^.

Premenopausal breast cancer is a distinct disease as the hormonal milieu is different from the postmenopausal women. Association of common risk factors such as obesity and waist circumference also differ from postmenopausal BC, some studies even suggest inverse relationship^8^. Young women also tend to have a higher proportion of aggressive subtypes such as  Human epidermal growth factor receptor 2 (HER2) amplified and triple negative breast cancer (TNBC) indicating difference in distribution of molecular subtypes^9,10^. Early detection of the BC is challenging in the premenopausal women due to high density of the breast tissue. Moreover, current therapeutic regimens are based on the menopausal status and molecular subtype of BC^11^ that contributes to differential prognosis and disease outcome between the pre and postmenopausal patients. Investigation into the trends of BC by menopausal status is important in countries with larger proportion of young premenopausal women from the public and patient health perspectives to inform prevention and early detection policies.

It has been an issue of debate if BC in Asia with occurrence at early age, is a different disease and examination of the population structure showed it could be due to strong cohort effect^12^. While the patterns of BC by menopausal status have been examined in broad geographical areas, information from regions such as individual countries and subregions is lacking. We evaluated the association of various reproductive and clinicopathological characteristics between pre and postmenopausal BC from a hospital-based cohort in India and examined the factors correlated with prognosis within them.

## Materials and methodology

### Patient cohort

This is an observational, retrospective, hospital-based study conducted at St. John’s Research Institute, Bangalore. In this study, 446 BC patients were recruited from two tertiary cancer care hospitals in Bangalore, India between the year 2008 and 2013. Patients included in this study were women diagnosed with primary BC tumors which were confirmed histologically. These patients were followed-up to 9 years, with a total loss to follow-up of less than 4% and a median follow-up duration of over 6 years. The study was approved by an individual institutional ethical review board. Informed consent was obtained from all the patients. All the clinical and histopathological information such as tumor size and location, grade of differentiation, lymphovascular invasion, perineural invasion, lymph node metastasis, treatment regimens, type of surgery was obtained from the patient’s clinical records. Information on disease progression such as local recurrence, distant metastases was collected during the regular follow up and duration to the event and survival period were recorded during the study.

Tumor cells showing ≥ 1% immunopositivity were considered as positive for estrogen receptor (ER) and progesterone receptors (PR) expression. HER2 expression data was obtained from the hospital records that were assessed by IHC and FISH for equivocal cases. Cases with 3+ IHC staining, or FISH amplified were categorized as HER2 positive. Based on expression of ER, PR and HER2 tumors were classified into three subtypes: hormone receptor positive (HR +), HER2 amplified and TNBC. Women were categorized into pre and postmenopausal groups based on age 50 years as cut-off^13^.

To validate our findings, two publicly available external databases, Sweden Cancerome Analysis Network-Breast (SCAN-B) and The Molecular Taxonomy of Breast Cancer International Consortium (METABRIC) were used. SCAN-B is the large population-based cohort of primary breast tumors. Our analysis included a cohort of 3207 primary BC patients from the SCAN-B study (GSE202203)^14^ and data was downloaded from Gene expression Omnibus database (GEO)^15^. TNBC patient’s data (n = 320) (as per the clinical groups mentioned in the dataset) was used for analysis. METABRIC data (EGAS00000000098) was downloaded from cBioPortal^16–18^. TNBC patient’s data (*n* = 307) (as per the 3 gene classifier subtype mentioned in the dataset) from this database was considered for analysis.

### Statistical analysis

Statistical analysis was performed using SPSS statistical software Version 25.0 (SPSS, Chicago, IL, USA). Descriptive statistics was used to examine the distribution of the study variables. Association of various reproductive and clinicopathological factors with pre and postmenopausal groups were assessed using Chi square test. Disease free survival (DFS) was calculated as duration from date of surgery till the first evidence of metastasis/recurrence. Overall survival (OS) was calculated as the duration from surgery to the death of the patient. Survival probability was calculated by Kaplan Meier survival analysis and was compared between groups using log rank test, in an entire cohort and stratified by menopausal status, the risk factor associated towards the progression of disease was calculated using univariate and multivariable Cox proportional hazard model. Results are represented as hazard ratio (HR) with 95% confidence interval (CI). In addition, stratified analysis was also carried out by subtype of tumor category to compare the disease-free survival and overall survival between pre and postmenopausal status. *P* value < 0.05 was considered as statistically significant.

### Ethical approval

The studies involving human participants were reviewed and approved by the Institutional Ethical Committee, St John’s Medical College and Hospital, Bangalore (No. IEC/1/655/2019). The patients/participants provided their written informed consent to participate in this study. This study was performed in line with the principles of the Declaration of Helsinki.

## Results

Median age of the cohort was 55 years, ranging between 24 and 88 years. There were 162 premenopausal (36.3%) and 284 postmenopausal (63.7%) BC patients. Large proportion of the patients were from urban background (80–85%), 12% were illiterate and 76% belonged to home maker category. Around 13% patients had family history of BC and 15% patients had family history of any other cancer in the first-degree relatives. There was an overlap of 3% cases having family history of BC and any other cancer in the first-degree relatives.

Duration of the lump felt was ranging from a week to few years. In majority of the cases duration of the lump felt was between > 1 month and 12 months (55%). There were three cases that were detected at screening. Of the total patients, 52% had tumor in the right breast, 45.5% had in the left breast and 2.5% had bilateral BC. Most common mode of detection was mammography (54%) followed by fine needle aspiration cytology (FNAC) (27%), ultrasound, biopsy/surgery, computed tomography (CT) scan and positron emission tomography (PET) CT. All patients received age and stage appropriate standard of care treatment. Majority of the patients (> 90%) received anthracycline or taxane based chemotherapy and all HR + patients received antihormonal therapy with tamoxifen or aromatase inhibitor. Most common site of metastasis was bone, liver, lung, followed by skeletal muscle, brain and others.

### Association of reproductive features between pre and postmenopausal breast cancer

The association of reproductive features like, age at menarche, age at first childbirth (FCB) and last childbirth (LCB) and parity with the risk of the BC development/ progression were assessed between pre and postmenopausal BC groups. Based on median cut-off of age at menarche, FCB and LCB and parity, BC patients were divided into two categories. Results are represented in Table1.

**Table 1:** Association of reproductive features between pre and postmenopausal breast cancer.

Variable	Subcategory (N = 446)	Premenopausal group (N = 162) n (%)	Postmenopausal group (N = 284) n (%)	*P* value
Age at Menarche (years)	< 14 (n = 193)	66 (45.2)	127 (48.7)	0.503
	≥ 14 (n = 214)	80 (54.8)	134 (51.3)	
Age at FCB (years)	< 23 (n = 169)	55 (42.6)	114 (48.1)	0.316
	≥ 23 (n = 197)	74 (57.4)	123 (51.9)	
Age at LCB (years)	< 30 (n = 138)	53 (56.4)	85 (42.7)	**0.029**
	≥ 30 (n = 155)	41 (43.6)	114 (57.3)	
Parity	< 2 (n = 54)	29 (21.1)	25 (10)	**0.003**
	≥ 2 (n = 330)	108 (78.9)	222 (90)	

*FCB* First childbirth, *LCB* last childbirth.

*p* < 0.05, Statistically significant (represented in bold).

We observed significantly higher proportion of postmenopausal BC women with older age at LCB (≥ 30 years) which was further supported by increased parity in this group. Age at menarche and FCB did not differ with menopausal status. We also did not find any association of family history of BC or any other cancer with pre and postmenopausal groups.

### Association of clinicopathological features between pre and postmenopausal breast cancer

Distribution of tumor characteristics such as tumor size (T size), lymph node (LN) status, grade, tumor infiltrating lymphocytes (TILs), did not differ between pre and postmenopausal groups. Premenopausal group had significantly higher proportion of ER negative (N = 162, premenopausal n = 69 (43%), postmenopausal = 93 (33%) (*p* = 0.038), HER2 amplified (N = 86, premenopausal n = 37 (23%), postmenopausal = 49 (17%) and TNBC tumors (N = 107, premenopausal n = 48 (30%), postmenopausal n = 59 (21%) (*p* = 0.012) compared to postmenopausal group. Association of various clinical characteristics of the tumor between pre and postmenopausal groups is represented in Table 2.

**Table 2:** Association of clinicopathological features between pre and postmenopausal breast cancer.

Variable	Subcategory (N = 446)	Premenopausal (N = 162) n (%)	Postmenopausal (N = 284) n (%)	*P* value
T size (cm)	T1 (n = 108)	39 (27)	69 (26)	0.860
	T2 (n = 253)	88 (60)	165 (63)	
	T3 (n = 49)	19 (13)	30 (11)	
LN status	Positive (n = 274)	105 (65)	169 (60)	0.268
	Negative (n = 172)	57 (35)	115 (40)	
Grade	I (n = 30)	9 (7)	21 (8)	0.525
	II (n = 186)	61(45)	125 (49)	
	III (n = 175)	66 (48)	109 (43)	
TILs	Present (n = 204)	81 (57)	123 (50)	0.136
	Absent (n = 185)	60 (43)	125 (50)	
ER	Positive (n = 284)	93 (57)	191 (67)	**0.038**
	Negative (162)	69 (43)	93 (33)	
PGR	Positive (n = 265)	93 (57)	172 (61)	0.514
	Negative (n = 181)	69 (43)	112 (39)	
HER2	Positive (n = 86)	37 (23)	49 (17)	0.353
	Negative (n = 307)	107 (66)	200 (70)	
	Equivocal (n = 53)	18 (11)	35 (13)	
Subtype	HR + (n = 253)	77 (47)	176 (62)	**0.012**
	HER2 (n = 86)	37 (23)	49 (17)	
	TNBC (n = 107)	48 (30)	59 (21)	

*LN status* lymph node status, *TILs* tumour infiltrating lymphocytes, *ER* estrogen receptor, *PGR* progesterone receptor, *HER2* human epithelial growth factor receptor, *HR* + hormone receptor positive, *TNBC* triple negative breast cancer.

*p* < 0.05, statistically significant (represented in bold).

### Association of clinicopathological features with disease progression within pre and postmenopausal tumors

The risk associated with each tumor characteristics towards the progression of the disease was evaluated by univariate Cox proportional hazard model in the entire cohort. Grade II and III tumors were categorised into high grade with grade I as the reference (low grade) for examination of association with survival. High grade (HR = 4.3 95% (1.06–17.5), larger tumor size (categorised as per AJCC criterion) (T3 vs. T1) (HR = 2.64, 95% CI = 1.43–4.86), LN positivity (HR = 2.9, 95% CI = 1.9–4.5), HER2 amplification (HR = 2.07 95% CI = 1.33–3.3) and TNBC subtype (HR = 1.62, 95% CI = 1.03–2.56) were significantly associated with increased hazard (*p* < 0.05), whereas presence of the TILs infiltration (HR = 0.62, 95% CI = 0.00–0.93), had significantly decreased hazard for the disease progression (*p* = 0.02). Pre and postmenopausal groups did not show any association with risk of the disease progression in our cohort. We further confirmed the association of these pivotal factors with progression of the disease by multivariate analysis. LN positivity, HER2 amplification and TNBC subtype were associated with significantly increased hazard whereas presence of TILs was associated with lower risk of disease progression in the entire cohort as mentioned in Table 3.

**Table 3:** Multivariate analysis of clinicopathological tumor characteristics in entire cohort, pre and postmenopausal groups for disease free survival.

	Reference	Variable	Entire cohort (N = 340)		Premenopausal groups (N = 119)		Postmenopausal groups (N = 221)	
			HR (95%CI)	*P* value	HR (95%CI)	*P* value	HR (95%CI)	*P* value
T size (cm)	T1	T2	1.2 (0.66–1.95)	0.673	0.67 (0.3–1.5)	0.33	1.7 (0.75–3.89)	0.65
	T1	T3	1.8 (0.86–3.47)	0.128	0.73 (0.2–2.7)	0.63	3.34 (1.2–8.8)	**0.016**
LN status	Negative	Positive	3.75 (2.16–6.59)	**0.0001**	3.0 (1.3–7.5)	**0.013**	4.65 (2.23–9.7)	**0.0001**
TILs	Absent	Present	0.54 (0.00–0.83)	**0.004**	0.49 (0.24–1.05)	**0.068**	0.66 (0.38–1.2)	0.140
Subtype	HR +	HER2 amplified	2.17 (1.33–3.55)	**0.002**	1.94 (0.92–4.13)	0.086	2.05 (1.06–3.99)	**0.032**
	HR +	TNBC	1.90 (1.1–3.27)	**0.022**	0.62 (0.22–1.73)	0.36	3.55 (1.84–6.83)	**0.001**

*HR* hazard ratio, *CI* confidence interval, *LN status* lymph node status, *TILs* tumor infiltrating lymphocytes, *HR* + Hormone receptor positive, *HER2* human epithelial growth factor receptor, *TNBC* triple negative breast cancer.

*p* < 0.05, statistically significant (represented in bold).

Next, we evaluated the association of these factors independently in the pre and postmenopausal groups. Among the postmenopausal tumors, association of tumor size, LN positivity, HER2 amplification and TNBC subtype showed the similar trends of association as in the entire cohort. Presence of TILs was associated with decreased hazard and LN positivity was associated with increased hazard of disease progression in premenopausal BC.

Though TNBC subtype was associated with higher hazard in the entire cohort and within postmenopausal subgroup, TNBC tumors within premenopausal group did not show any association with the hazard in multivariate analysis (Table 3). To verify these findings further, we evaluated the hazard associated with menopausal status within TNBC tumors alone. Both univariate (HR = 0.28 (0.0–0.67), *p* = 0.004) and multivariate analysis (HR = 0.20 (0–0-0.56), *p* = 0.002) showed within TNBC subtype, premenopausal BC patients had lower hazard of disease progression.

Kaplan Meier survival analysis confirmed the results of multivariate analysis and showed no difference in the DFS between pre and postmenopausal BC (mean survival time 77.1 vs. 77.9 months) (*p* = 0.77). We next examined the influence of subtypes on DFS independently within pre and postmenopausal BC. In postmenopausal BC, patients with TNBC and HER2 amplified tumors were associated with poor outcome as expected and there was no difference in the survival between patients with HER2 amplified and TNBC tumors (mean survival, HR + vs. TNBC, 83.8 vs. 54 months, *p* < 0.0001, HR + vs. HER2 amplified tumor 83.8 vs. 63.3, *p* = 0.014, HER2 amplified vs. TNBC 63.3 vs. 54 months, *p* = 0.328).

**Figure 1:**
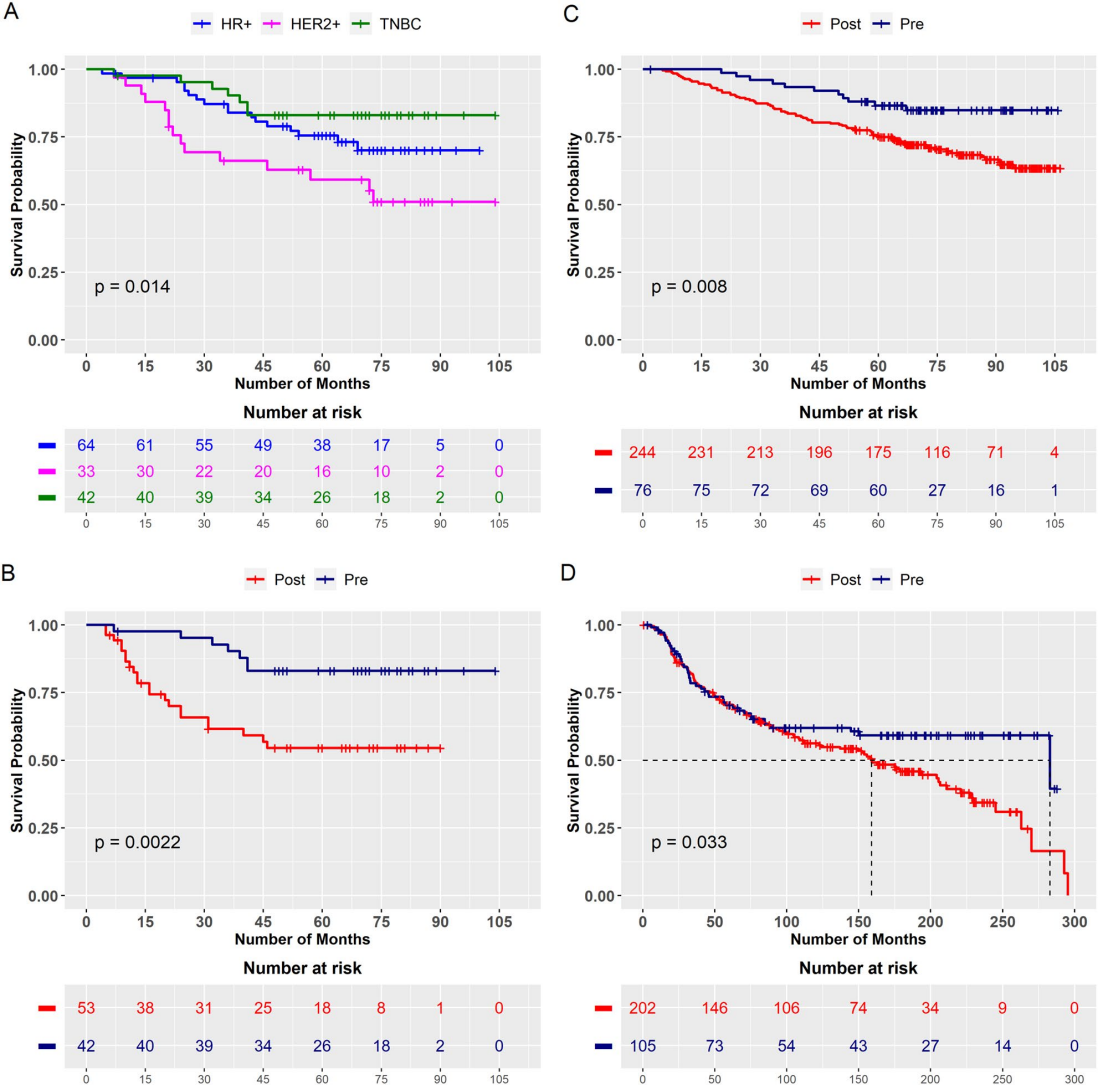
Kaplan Meier survival plots. (**A**) Disease free survival (DFS) across three subtypes in premenopausal breast cancer. (**B**) DFS in TNBC tumors between pre and postmenopausal BC. (**C**) Overall survival (OS) in TNBC tumors between pre and postmenopausal BC in SCAN-B database and (**D**) OS in TNBC tumors between pre and postmenopausal BC in METABRIC database.

However, comparison of DFS among different subtypes within premenopausal patients showed, no difference in the survival between HR + and TNBC breast cancer patients and both subtypes were associated with better outcome compared to patients with HER2 amplified tumors (Fig. 1A, mean survival, TNBC vs. HER2 amplified BC 79.2 vs. 61 months, *p* = 0.006. mean survival HR + vs. HER2 amplified BC, 74.4 vs. 61 months, *p* =0.058. mean survival, TNBC vs. HR + tumors 79.2 vs. 74.4 months, *p* = 0.228).

Next, we examined the DFS and OS independently in each of the molecular subtypes that is HR + , HER2 amplified and TNBC between pre and postmenopausal tumors. There was no difference in the survival between the two groups in the HR + tumors (mean survival, pre vs post 74.4 vs. 83.8 months, *p* = 0.374) and in HER2 amplified tumors (mean survival, pre vs. post 61 vs. 63.3 months, *p* = 0.690). No difference was observed in OS either.

Interestingly in the TNBC subtype, premenopausal patients were associated with better outcome compared to postmenopausal BC patients (Fig. 1B, mean survival, pre vs. post 79.2 vs. 54 months, *p* = 0.002) which was contrary to the notion that TNBC tumors are always associated with aggressive behaviour. Similar trends were noted with overall survival (mean survival, pre vs. post, 72.5 vs. 49.5 months, *p* = 0.002). To rule out if these results could be due to the bias within our cohort, we validated our findings in two publicly available larger databases, SCAN-B and METABRIC with different population. OS analysis in these cohorts confirmed our findings on association of premenopausal BC patients with better prognosis within TNBC subtype (Fig. 1C,D, In SCAN-B database, mean survival, pre vs. post 94.2 vs. 84.4 months, *p* = 0.008 and in METABRIC database, mean survival, pre vs. post, 186.8 vs. 157.2 months, *p* = 0.033). We also performed the COX proportional hazard ratio model in these two cohorts to verify similar trends in pre and postmenopausal groups within TNBC subtype of tumors. In both SCAN-B and METABRIC datasets, TNBC tumors with premenopausal BC were associated with decreased hazard on univariate analysis [SCAN-B, HR = 0.42, 95% CI = 0.00–0.82), *p* = 0.010, METABRIC, HR = 0.68, 95% CI 0.00–0.98), *p* = 0.034] and multivariate analysis (SCAN-B, HR = 0.45, 95% CI = 0.00–0.85), *p* = 0.014, METABRIC, HR = 0.56 (0.00–0.82), *p* = 0.003) compared to postmenopausal tumors.

## Discussion

Epidemiological studies have often distinguished between pre and postmenopausal BC mainly based on difference in risk factors like age and reproductive factors. Risk factors such as abdominal obesity, parity and age at childbirth are shown to be different between pre and postmenopausal BC^19–21^. Average age of women with BC in developing countries is about 10 years lower than developed nations, leading to higher burden of younger patients. As early onset of disease could reflect more fundamental etiologic differences, we investigated the different reproductive and clinical features and compared them between pre and postmenopausal BC.

Previous studies have evaluated the association and prognostic significance of reproductive features such as age at menarche, FCB, LCB and parity with breast cancer outcomes^22,23^. In our study cohort, women in postmenopausal age group had significantly higher age at LCB and higher parity. Early age at menarche, older age at menopause, older age at FCB and LCB are known to increase women’s risk of developing BC^24^. Zhang et al. evaluated the association of multiple reproductive factors with BC prognosis with respect to menopausal status and reported association of increased hazard with older age at FCB and LCB in premenopausal BC patients^10^. Significant inconsistencies are however found in association of the reproductive factors with prognosis in both pre and postmenopausal BC. A prospective cross-sectional study conducted in the Indian BC patient’s cohort reported that premenopausal BC patients had younger age at menarche, older age at FCB, lesser parity, lesser BMI and denser breast tissue compared to postmenopausal BC patients^25^. A study by Butt et al. reported that most of risk factors for pre and postmenopausal BC are same except less parity, which increased the risk for postmenopausal BC^21^.

In tune with the previous reports, our cohort of premenopausal BC had higher proportion of ER negative, HER2 amplified and TNBC tumors compared to postmenopausal tumors which were predominantly hormone receptor positive (9, 28, 2). There was no difference in the relative distribution of other clinicopathological features between pre and postmenopausal groups. Other studies^26,27^ on premenopausal tumors have shown difference in tumor variables such as increased tumor size, lymph node metastasis, ER, PR and Ki67 expression and decreased HER2 and p53 expression compared to postmenopausal tumors. We did not find these differences between the pre or postmenopausal BC patients in our cohort. These differences might be due to different ethnicity as well as proportion of pre and postmenopausal patients in these cohorts compared to our study cohort. Premenopausal patients in these cohorts were proportionately much higher than in our study.

Among the molecular subtypes, previous studies such as the Carolina breast cancer study has reported premenopausal BC patients have higher prevalence of Basal-like breast tumors compared with postmenopausal patients^28^. The well-established clinical factors such as LN involvement, HER2 amplified status showed increased hazard of poor prognosis as expected. Better survival observed in the premenopausal TNBC tumors is unique finding of our study. To nullify the effect of inadequate chemotherapeutic treatment, which might influence adverse prognosis, we verified and confirmed that all the patients had received stage appropriate standard of care with anthracyclins and taxane based regimens. Multiple studies have reported higher proportion of aggressive subtype TNBC within Asian countries^29,30^. A very recent study by Nishimura et al. evaluated the clinical significance of menopausal status in TNBC. This study reported that higher proportion of TNBC tumors in postmenopausal patients and their association with favourable tumor characteristics and better DFS compared to premenopausal patients^31^ which is contrary to our observations. Disparities in TNBC outcomes have been reported based on race and ethnicity in multiple studies earlier^32–34^. Younger median age of our cohort and ethnicity-based differences between Indian and Japanese women might have contributed to the observed difference. Recent study by Yunzi et al.^35^ investigated tumor characteristics and mortality in south east Asian (SEA) women by regional distribution specific to countries in SEA and found the cancer specific survival was best for Japanese women among all SEA ethnic populations and suggests disaggregation by country or region of origin to identify subgroups that are at risk for worse outcomes.

TNBC tumors are heterogenous and consists of multiple molecular subtypes. PAM 50 molecular subtyping has shown inclusion of normal breast-like and claudin-low molecular subtypes other than basal-like tumors. Normal breast-like tumors have been clustered with basal-like tumors in the majority of the studies, but with better prognosis^36^. TNBC in the premenopausal age group may be enriched with normal breast like TNBC than basal like TNBC tumors, however there are very few studies to examine or compare prognostic significance of premenopausal TNBC tumors. Our findings need further validation in large cohorts with diverse ethnicity for confirmation.

Our study has limitations due to the retrospective cohort, with non-availability of information on weight, BMI and obesity amongst the all the women enrolled for the study. Previous studies have correlated the importance of waist circumference and central obesity as both risk factor for BC occurrence and prognosis^37^. Excess weight gain with onset of menopause among women with BC is also associated with other morbidities. We were unable to derive association of prognosis with density of the breast tissue due to lack of radiological information which is considered significant risk factor especially among young premenopausal women^38^. Lack of therapeutic interventions for ovarian suppression among the premenopausal women could have influenced the prognosis in this subgroup though majority of the women were treated with standard of care prevalent at the time of diagnosis. Findings from recent trails such as TEXT and SOFT^39^ showing improvements with use aromatase inhibitors along with ovarian suppression over the traditionally used tamoxifen is likely to influence the prognosis among young patients with BC in future.

In conclusion, findings of our study confirm the higher proportion of aggressive subtypes such as ER negative, HER2 amplified and TNBC within premenopausal BC suggesting the effect of hormonal milieu on the biology of tumor development. Parity and age at last childbirth were different between pre and postmenopausal women. We also noticed differential prognosis in TNBC tumors based on menopausal status in our cohort, which needs verification based on ethnicity and age distribution across other cohorts. Categorising tumor subtypes especially, TNBC tumors based on the menopausal status will help in developing better treatment strategies and in predicting outcome.

## Data availability

The datasets generated during and/or analysed during the current study are available from the corresponding author on reasonable request. Publicly available datasets used in the current study: Sweden Cancerome Analysis Network-Breast (SCAN-B) (GSE202203; https://www.ncbi.nlm.nih.gov/geo/query/acc.cgi?acc=GSE202203). The Molecular Taxonomy of Breast Cancer International Consortium (METABRIC). (EGAS00000000098; https://cbioportal-datahub.s3.amazonaws.com/brca_metabric.tar.gz).
